# Neoadjuvant therapy with anlotinib in a locally advanced and pulmonary metastasis PTC patient harboring TERT promoter and BRAF^V600E^ mutations: a case report

**DOI:** 10.20945/2359-3997000000659

**Published:** 2023-06-19

**Authors:** Yan-Jun Su, Shao-Hao Cheng, Jun Qian, Ming Zhang, Wen Liu, Xiang-Xiang Zhan, Zhu-Quan Wang, Hai-Dan Liu, Xing-Wei Zhong, Ruo-Chuan Cheng

**Affiliations:** 1 The First Affiliated Hospital of Kunming Medical University Department of Thyroid Surgery Kunming China Department of Thyroid Surgery, The First Affiliated Hospital of Kunming Medical University, Kunming, Yunnan, P.R. China; 2 The First People’s Hospital of Honghe State Department of General Surgery Mengzi China Department of General Surgery, The First People’s Hospital of Honghe State, Mengzi, PR. China

## Abstract

A 71-year-old woman with recurrent papillary thyroid carcinoma (PTC) was referred to our hospital. A computed tomography scan revealed extensive recurrence in the neck, invading sternocleidomastoid muscle, internal jugular vein, sternal end of the clavicle, strap muscle and skin; and lateral compartment and subclavian lymph nodes were also involved. Multiple pulmonary micrometastases also noticed. The tumor was considered unresectable; however, the patient was unwilling to accept highly invasive surgery. Therefore, we initiated neoadjuvant therapy with anlotinib, 12mg p.o. daily with a 2-week on/1-week off regimen. The tumor shrunk to resectable state after 4 cycles of treatment, and after 3 weeks of withdrawal, successful surgical resection without gross tumor residual was performed. Pathology confirmed as classic PTC harboring coexistent TERT promoter and BRAF^V600E^ mutations by NGS. After anlotinib therapy, apoptosis induction was observed, and proliferation increased, which was due to three weeks of anlotinib withdraw. Structual recurrence was recorded at 6 months after operation due to no further treatment was taken. Our finding suggests that anlotinib could represent as a good treatment option for patients with locally advanced (with or without distant metastasis) PTC; Anlotinib treatment resulted in sufficient reduction of the tumor mass to enable total thyroidectomy and radioactive iodine treatment, providing long-term control of the disease.

## INTRODUCTION

Thyroid cancer incidence has been rising over the last decades worldwide (1-2), papillary thyroid carcinoma (PTC) accounts for more than 80%-90% of all thyroid malignancies. Although most of PTCs are successfully managed with a multimodal approach, incorporating surgical resection, followed by radioactive iodine (RAI) therapy and thyroid stimulating hormone (TSH) suppression therapy. Few patients were locally advanced at initial presentation or developed into inoperable state after recurrence. Advanced tumor stage with infiltration of the surrounding structures and organs is the strongest prognostic factor. Surgery is still considered to be the main treatment for locally advanced thyroid cancer (3). To achieve surgical clearance in patients with locally advanced tumors, vital structures may need to be sacrificed, leading to both functional impairment and cosmetic deformity. Failure to control local disease due to invasive cancer is one of the main risk factors for recurrence or mortality (4).

Neoadjuvant therapy has been successfully used in the treatment of other advanced tumors. PTC is insensitive to traditional chemotherapy, and targeted kinase inhibitors have been increasingly utilized in the treatment of radioactive iodine-refractory differentiated thyroid cancer (RAIR-DTC). However, due to surgical technology, lack of RAI treatment or economic reasons, some patients with advanced PTC have no chance to develop into iodine refractory state. Lenvatinib (5,6), sorafenib (7) and apatinib (8) were reported to have neoadjuvant role in selected cases of locally advanced PTC to reduce tumour volume and therefore subsequent surgical resection. Anlotinib has shown strong therapeutic effect on RAIR-PTC and manageable adverse effects (9).

Herein, we report a case of 71-year-old women who presented with locally advanced and multiple pulmonary metastatic PTC following initial operation. She was treated with 4 cycles of anlotinib, which rendered her locoregional disease resectable and subsequent reoperation was performed. And the tumor recurred 6 months after the operation due to no further anti-tumor treatment. Lessons learned from this case are discussed.

## CASE PRESENTATION

### Treatment prior to anlotinib

A 71-year-old woman came to the First Affiliated Hospital of Kunming Medical University in April, 2020 because of thyroid cancer recurrence for more than 2 years. She was initially diagnosed as PTC when she was 67 years old in August, 2017 and treated in another tertiary hospital, including right thyroid lobectomy, isthmusectomy, left thyroid subtotal lobectomy and prophylactic unilateral level VI dissection. The postoperative pathological stage was pT3bN0M0, II.

**Figure 1:**
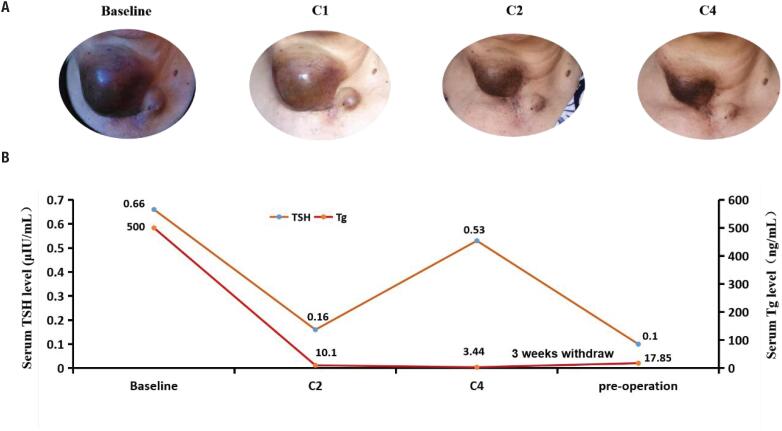
Changes of tumor appearance and serum Tg, TSH level following anlotinib therapy. (**A**) Changes of tumor appearance from baseline to C1, C2 and C4. (C1, C2, C3: refers to the cicles of anlotinib treatment). (**B**) Changes of serum Tg, TSH level following anlotinib therapy from baseline to C2, C4 and pre-operation.

**Figure 2:**
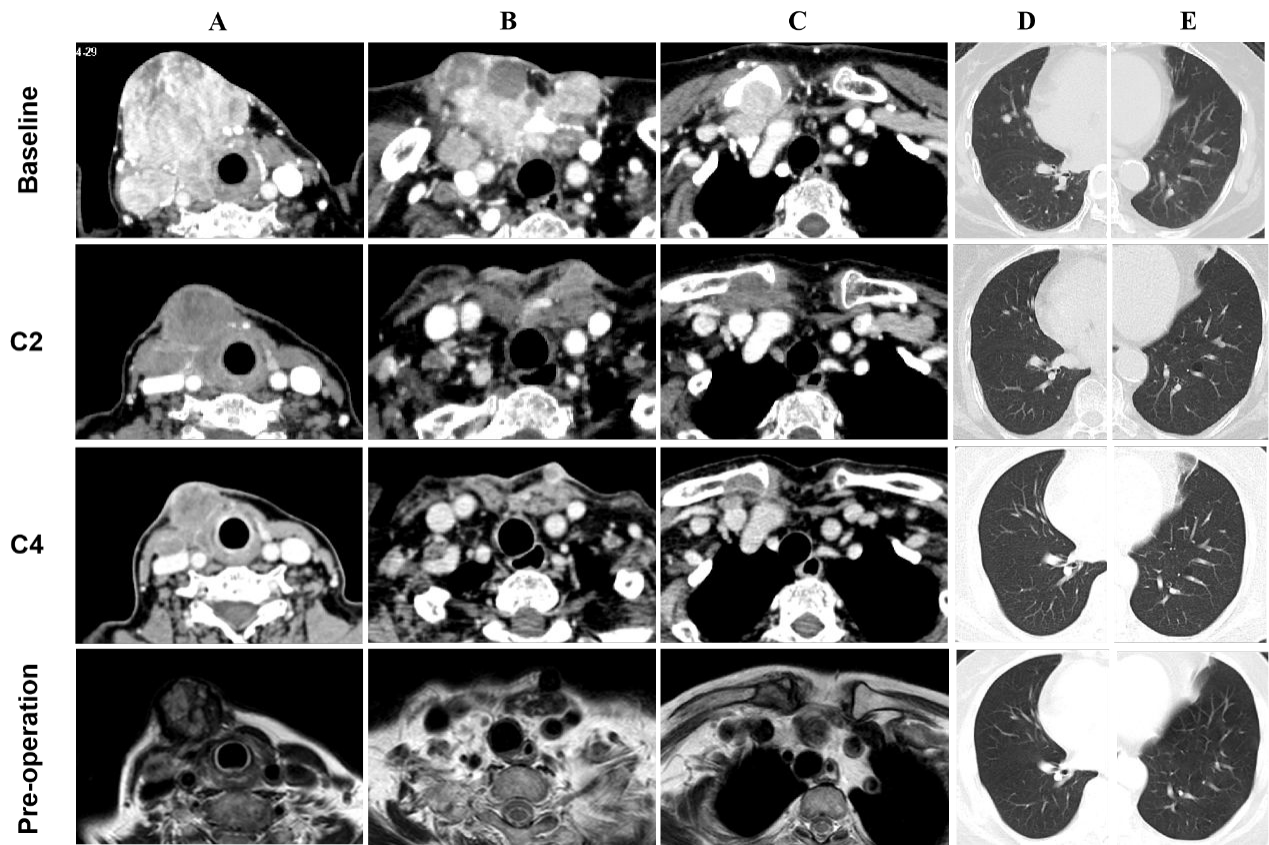
Imaging evaluation on tumor shrinkage following anlotinib therapy CT (Baseline, C2, C4); MRI (pre-operation). **A:** Recurrent tumor within thyroid bed. **B:** Recurrent tumor in the neck root in front of trachea. **C:** Clavicle and subclavian lymph nodes. **D:** Metastatic lesion in the right lung. **E:** Metastatic lesion in the left lung.

TSH suppression therapy with L-T4 was applied, but RAI therapy was not recommended. Recurrence was revealed by ultrasound within thyroid bed at 5 months after surgery, and the patient refused reoperation and was treated with non prescription Chinese medicine. The recurrent tumor in the neck grown rapidly within 2 months before coming to our hospital, presented with local pain, burning sensation and skin color change (Figure 1A – baseline). A contrast-enhanced CT scan (Figure 2 – baseline) showed extensive recurrence in the neck, invading sternocleidomastoid muscle, internal jugular vein, sternal end of the clavicle, strap muscle and skin; and lateral compartment and subclavian lymph nodes were also involved. Multiple pulmonary micrometastases also demonstrated. After MDT discussion, it was considered that the recurrent tumor could not be treated by R0 (represents complete resection of the tumour and negative microscopic margins) or R1 (The tumour is seen to be cleanly removed with the naked eye, but when viewed under the microscope, the tumour cells are visible at the cut edge) resection. This patient was unwilling to perform highly invasive surgery. Based on the excellent response of RAIR-DTC following anlotinib therapy (partial response was achieved at 2 cycles), the patient was willing to try the neoadjuvant treatment with anlotinib.

### Neoadjuvant therapy with anlotinib

On May 8, 2020, anlotinib was administered orally at 12 mg/day for 2 weeks, followed by 1 week off treatment. After treatment with anlotinib, the tumor size decreased significantly (Figure 1A). The local pain and burning sensation were relieved after 1 week of treatment. The CT evaluation after 2 cycles of treatment (Figure 2 C2) demonstrated a remarked reduction in tumour volume compared with baseline from 60 × 52 × 54 mm to 55 × 42 × 32 mm, and the pulmonary metastases also significantly shrunk. Due to adverse effects such as hand-foot syndrome and hypertension, anlotinib was reduced to 10 mg in 4^th^ cycle. A CT scan was repeated and the tumor was significantly shrunk and the pulmonary lesions were basically disappeared after 4 cycles of treatment. Anlotinib was stopped for 3 weeks and subsequent reoperation was performed. A preoperative whole body bone imaging showed osteolytic destruction of the right clavicle near the sternoclavicular joint and active bone metabolism, indicating bone metastasis. Serum Tg also decreased following anlotinib therapy (Figure 1B). Preoperative MRI examination showed tumor enlarged slightly, which was consistent with the increase of serum Tg with 3 weeks of withdraw.

### Surgery following anlotinib therapy

**Figure 3:**
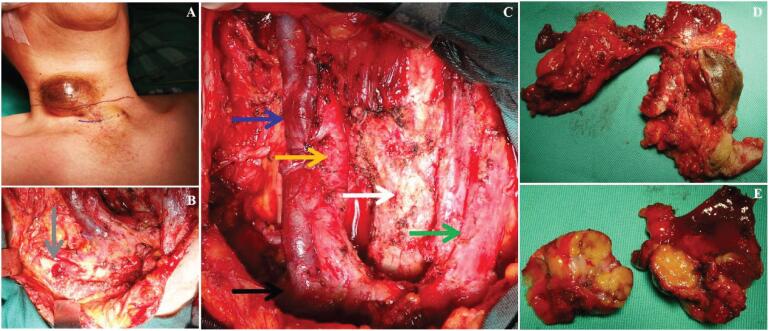
Intraoperative findings during surgery. **A:** Photo taken before incision was made. **B-C:** Photos taken after tumor resection, vital structures are showed with arrows (**Blue:** Internal jugular vein; **Yellow:** Common carotid artery; **White:** Trachea; **Green:** Sternocleidomastoid muscle on the left; **Black:** Junction of subclavian vein and internal jugular vein; **Gray:** Lateral end of clavicle. **D-E:** The appearance and profile of the resected specimen.

The scope of surgery includes resection of the recurrent tumor in the right thyroid bed, functional neck dissection in right neck (internal jugular vein was preserved and the accessory nerve and sternocleidomastoid muscle was removed), the right medial part of the clavicle was resected and the subclavian lymph nodes were cleaned, bilateral central neck dissection (CND) was performed, and the involved skin and strap muscles were also resected. Encouragingly, no tumor infiltration of trachea, larynx and cervical sheath blood vessels were observed, and no inflammatory reaction or scar adhesion were found during the operation. R1 resection without gross tumor residue was achieved. However, it is a pity that total thyroidectomy was planned before operation, but intraoperative nerve monitoring revealed right recurrent laryngeal nerve injury during CND, and the left residual thyroid tissue was preserved (Figure 3).

### Pathology and molecular analysis

We reviewed the pathology of primary tumor from the first operation and confirmed to be classic PTC, which was consistent with that of the second operation. Lymph nodes were involved in bilateral central compartment, sub-level IIa and level III. The right clavicle was also involved. Cell proliferation and apoptosis in tumor tissues were tested. TUNEL staining showed increased apoptosis after anotinib therapy, especially in recurrent tumor within thyroid bed and lymph node. Interestingly, Ki67 immunohistochemical staining showed increased proliferation when second operation was performed with three weeks of anlotinib withdraw compared with in first operation. CD31 immunolabeling was assessed, and observed no reduction in the number and caliber of vessels within fibrovascular cores after anlotinib therapy.

Tumor-specific genetic changes using next-generation sequencing (NGS) panel covers 50 genes was performed for the 2^nd^ surgical tissue excised from thyroid bed. The results showed the TERT p.C228T mutation and c.T1799A: p.V600E mutation of BRAF gene.

### Follow up and outcome

TSH suppression therapy was performed with target TSH ≤ 0.01 μIU/mL. The patient was not treated with RAI due to subtotal lobectomy was performed in left thyroid lobe, and molecular analysis revealed coexistent TERT promoter and BRAF^V600E^ mutations. Although continued treatment with anlotinib was suggested, she did not take it. Thyroid function was measured at 6 weeks after operation, the suppressed Tg was 11.62 ng/mL (with negative anti-Tg antibody), which was attributed to the residual thyroid and no further treatment was given.

Structural lesions were observed in the neck by an enhanced CT scan at 6 months after operation (Figure 5A). A chest CT showed that most of the metastatic tumors disappeared after anlotinib therapy did not reappear, while some regrew, and no new lesions were noticed (Figure 5B). Meanwhile, serum Tg under TSH suppression increased to 80.46 ng/mL, which also indicated recurrence. Since then, this patient continued to take anlotinib, 12 mg p.o. daily. At the 1-year follow-up evaluation, no tumor recurrence or metastasis was observed.

**Figure 4:**
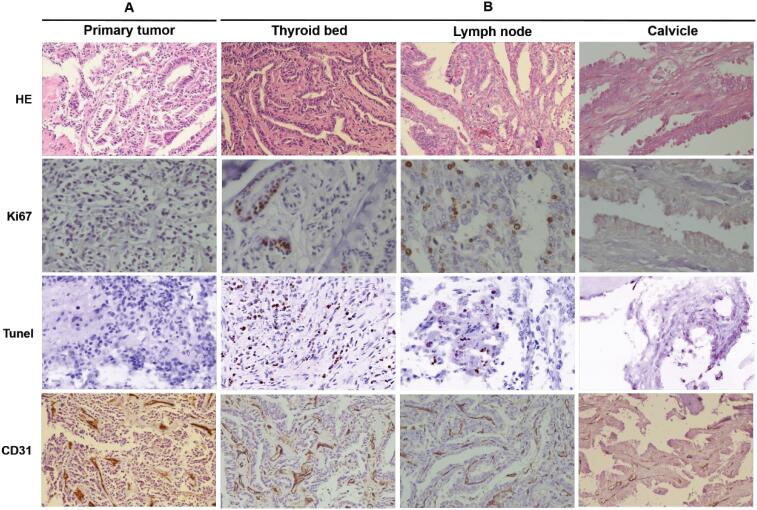
Histopathological analysis of the surgical specimens. **A**: Pre-neoadjuvant therapy (specimen from 1^st^ operation). **B:** Post-neoadjuvant therapy (specimen from 2^nd^ operation), including recurrent tumor within thyroid bed, lymph node and clavicle (HE and CD31: 200 ×, Ki67 and TUNEL: 400 ×).

**Figure 5:**
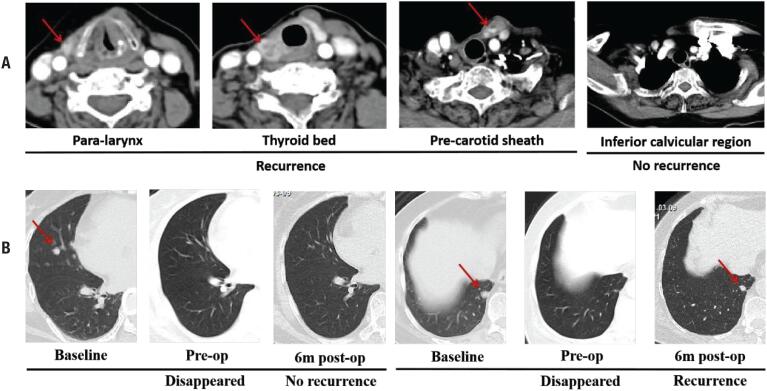
Imaging evaluation at 6 months after operation following anlotinib therapy.

## DISCUSSION

Surgical resection remains the mainstay of treatment for locally advanced differentiated thyroid cancer (3,10), however, failure to control local disease is one of the main risk factors for recurrence or mortality (4). Radioiodine (RAI) has been applied in the postsurgical treatment of DTC for years. The role of RAI in locally advanced DTC, however, is matter of debate (11). Only those who developed into refractory radioiodine state were indicated for kinase inhibitor therapy. Limited treatment options is available for those who have no chance to develop into iodine refractory state due to lack of surgical technique or RAI treatment or other reasons. DTC is insensitive to traditional chemotherapy, the current DTC guidelines do not recommend neoadjuvant therapy for patients with unresectable tumor in whom surgery is contraindicated (12).

In this case we report, tumor relapsed 5 months after initial surgery, indicating highly invasiveness nature of the tumor coexisting TERT promoter and BRAF^V600E^ mutations. This patient refused reoperation or RAI therapy, and treated with traditional Chinese medicine. Only when tumor progressed into inoperable state, leading to serious clinical symptoms, did this patient seek for effective treatment. It is conceivable that the infiltrated skin rupture would cause massive bleeding. Meanwhile, great vessels, laryngeal and trachea infiltration, lung metastasis can also lead to serious outcome. Effective treatment is imminent for this patient.

Many tyrosine kinase inhibitors (TKIs) are increasingly being used to treat RAIR-DTC; and lenvatinib and sorafenib were approved by the U.S. Food and Drug Administration to be used in RAIR-DTC. Choice of those two targets drugs is likely to depend on an individual patient’s circumstances (13). TKI as neoadjuvant therapy for locally advanced PTC have been documented in 6 case reports (The electronic databases PubMed/Medline and Embase were searched for studies of neoadjuvant therapy for thyroid carcinoma), including 3 cases with lenvatinib (5-6,14), 2 cases with sorafenib (7,15) and 1 case with apatinib (6 weeks) (8); Tumor reduction was observed in all cases, allowing subsequent surgery performed (Table 1). Those results indicate TKIs as an effective and feasible alternative for unresectable locally advanced PTC. In current case, anlotinib was used as neoadjuvant therapy for 3 months, which was shorter than lenvatinib (16 weeks to 14 months) and sorafenib (6 months to 13 months) as neoadjuvant therapy, dramatic shrinkage was achieved and micro-pulmonary metastases disappeared, and subsequent resection was performed.

**Table 1:** A table summarizing previous reports of initially unresectable DTC cases

Years	Authors	Patients	Age	Clinical pathological characteristics	Drugs	Dose	Length of treatment	Adverse drug events	Outcomes
2017	Tsuboi and cols. (5)	Man	73	Papillary Thyroid Carcinoma (PTC) T4a N1b M0, stage IVA	Lenvatinib	Total dose, 966 mg for 22 weeks	22 weeks	Grade 3 proteinuria and hypertension	No distant metastasis
2019	Stewart and cols. (6)	Women	73	Papillary Thyroid Carcinoma (PTC) pT4a N0 R1	Sorafenib Lenvatinib	400 mg twice daily 24 mg daily	1 months 13 months (Total 14 months)	Gastro-intestinal side effects Weight loss, stomatitis, and loose stool	No distant metastasis
2020	Iwasaki and cols. (14)	Women	75	Papillary Thyroid Carcinoma (PTC)	Lenvatinib	14 mg per day	4 months	2 HT, grade 2 hand-foot syn-drome, and grade 2 anorexia	The patient is alive three months after surgery, and lung metastases have disappeared on CT images.
2018	Danilovic and cols. (7)	Man	20	Papillary Thyroid Carcinoma (PTC), pT4pN1bM1	Sorafenib	400 mg twice daily	13 months	Hand and foot skin reaction, and diarrhea	No distant metastasis
2019	Nava and cols. (15)	Man	32	Papillary Thyroid Carcinoma (PTC) pT4a N1b Mx – stage I	Sorafenib	800 mg per day	6 months	Hypertension, and grade II hand-foot syndrome	One-year post-surgery the patient is asymptomatic with a status of disease defined as an incomplete biochemical response.
2021	Zhang and cols. (8)	Women	64	Papillary Thyroid Carcinoma (PTC) pT4aN1aM0, stage III	Apatinib	500 mg orally once a day	6 weeks	Mild hypertension	At the 1-year follow-up evaluation, no tumor recurrence or metastasis was observed

Anlotinib might be a novel therapeutic option for patients with advanced PTC. Anlotinib is an oral TKI that was originally designed to inhibit VEGFR2/3, FGFR1-4, PDGFRα/β, c-Kit, and Ret (16,17), thereby exerting inhibitory effects on tumor angiogenesis and growth (18), tumor invasion (19), lymphangiogenesis and lymphatic metastasis (20). A previous study showed that anlotinib had stronger anti-angiogenic activity than three other angiogenesis inhibitors, sorafenib, sunitinib, and nintedanib (21). Preclinical studies have shown that anlotinib inhibits the cell viability of PTC, and suppresses the migration of thyroid cancer cells in vitro and the growth of xenograft thyroid tumors in mice (22). In addition, an ongoing phase II trial (NCT02586337) evaluating the efficacy and toxicity of anlotinib in patients with RAIR-DTC showed excellent response rate (unpublished data). We observed partial response with 2 cycles of anlotinib treatment, and sharply serum Tg decrease was observed in 1 patient in this trial (9). Significant tumor reduction and serum Tg decrease also noticed in current patient. Excellent structual and serological response in this patient suggest anlotinib would be an effective therapeutic strategy for patients with advanced PTC.

Anlotinib exhibits efficacy in various solid tumors; however, predictive biomarkers for anlotinib remain unclear. Anlotinib plays important roles in intracellular tyrosine phosphorylation and intracellular signaling (21). It is necessary to find ideal biomarker for predicting efficacy so as to seek patient population. Tan and cols. (23) recently demonstrates TERT promoter mutation governs BRAF-mutant cancer cells apoptotic and the genetic duet of BRAF^V600E^ and TERT promoter mutations represents an Achilles Heel in cancer for effective therapeutic targeting. After anlotinib treatment, apoptosis induction was observed not only in recurrent tumor within thyroid bed, but also in metastatic lymph nodes. We observed increased proliferation after three weeks of anlotinib withdraw, which was consistent with slightly regrew by preoperative MRI evaluation. We previously reported anlotinib treatment in 1 RAIR-PTC patient harboring TERT promoter and BRAF^V600^E mutations, and significant reduction of target lesions of any site was noticed (9). This patient also carried TERT promoter and BRAF^V600E^ mutations with excellent response to neoadjuvant therapy with anlotinib, which again suggest coexistent TERT promoter and BRAF^V600E^ mutations might be a biomarker to predict the beneficial effect of anlotinib in PTC, and further study is needed.

### Limitations

Although satisfactory response was achieved in this patient with anlotinib as neoadjuvant therapy, 2 limitations also exit. Firstly, the unresected thyroid tissue of left lobe during the second operation rendered serum Tg could not be used as an effective tumor marker for recurrence monitoring. Secondly, RAI was applied in patients with locally advanced PTC patient following neoadjuvant therapy with TKIs and subsequent surgery (5-8,14-15). The retained thyroid tissue became a stumbling block in the treatment of RAI in this patient, and continued anlotinib treatment was suggested after operation, but the patient did not, resulting in structural recurrence in the neck and pulmonary metastases regrowth 6 months after surgery. Thirdly, we observed tumor regrowth and increased proliferation with three weeks of anlotinib withdraw, which can partly explain the increased proliferation. Finally, we observed no difference in the number and caliber of vessels, this may partly be due to long time of withdraw. For TKIs therapy, drugs usually administered orally at different dosage for 2 weeks, followed by 1 week off treatment. Those pathological analysis results maybe different if operation was performed within one week after stop anlotinib. Lessons learned from this patient is that pulmonary micrometastases can not be successfully managed by 4 cycles of anlotinib therapy, and RAI or anlotinib is still needed to control local recurrence and distant metastases regrowth after surgery following neoadjuvant therapy.

In conclusion, our finding suggests that anlotinib could represent as a good treatment option in patients with locally advanced (with or without distant metastasis) PTC. Anlotinib treatment resulted in sufficient reduction of the tumor mass to enable total thyroidectomy and radioactive iodine treatment, providing long-term control of the disease. In addition, Anlotinib exhibits inhibitory effects on tumor angiogenesis and proliferation, and apoptosis induction. Coexistent TERT promoter mutation and BRAF^V600E^ mutation might be a biomarker to predict the beneficial effect of anlotinib. It is very encouraging to perform successful surgical resection after neoadjuvant therapy, but for better long-term outcome, it is necessary to continue anlotinib treatment or RAI following surgery. Further studies with large sample are needed to confirm the role of anlotinib as neoadjuvant therapy in patient with locally advanced PTC.
